# Impact of different fibrin glue application methods on inguinal hernia mesh fixation capability

**DOI:** 10.1038/s41598-024-63682-9

**Published:** 2024-06-04

**Authors:** Yoshitaka Takegawa, Norifumi Tsutsumi, Kazunori Yamanaka, Yuki Koga

**Affiliations:** 1 Medical Affairs Section, Research and Development Division, KM Biologics Co., Ltd., 1-6-1 Okubo, Kita-ku, Kumamoto-shi, Kumamoto Japan; 2 Department of Surgery, Munakata Medical Association Hospital, Fukuoka, Japan; 3 Nonclinical Development Section, Non-Clinical Study Department, KM Biologics Co., Ltd., Kumamoto City, Kumamoto Japan

**Keywords:** Gastroenterology, Medical research

## Abstract

The use of fibrin glue for inguinal hernia mesh fixation has been suggested to be effective in preventing hematomas and reducing postoperative pain compared to tacks and sutures.. The effect of fibrin glue can vary significantly based on the device used. This study assessed the efficacy of fibrin glue based on the type of devices used in an ex vivo system. The rabbit’s abdominal wall was trimmed to a size of 3.0 × 6.0 cm and was secured at the edges with metal fixtures. To measure the maximum tensile strength at the point of adhesion failure, the hernia mesh was fixed to the rabbit’s abdominal wall using fibrin glue in a 2 cm square area, left for 3 min, and then pulled at a speed of 50 cm/min. The test was conducted 10 times for each group. The median (minimum–maximum) tensile strength values using the spraying, two-liquid mixing, and sequential layering methods were 3.58 (1.99–4.95), 0.51 (0.27–1.89), and 1.32 (0.63–1.66) N, respectively. The spraying method had predominantly higher tensile strength values than the two-liquid mixing and sequential layering methods (*P* < 0.01). In conclusion, in hernia mesh fixation, the spraying method can be adopted to achieve appropriate adhesive effects.

## Introduction

Inguinal hernia repair using a mesh is widely performed for conditions such as inguinal hernia and abdominal wall incisional hernia. Then, it became a standard procedure for various cases. Over the years, there has been a continual development in surgical techniques, types of mesh, and fixation methods. Recently, hernia repair via mesh surgery itself has become safer and is associated with recurrence-free. Therefore, there has been a shift in research focus toward reducing complication rates, which led to an increased interest in less invasive methods.

In open inguinal hernia operations, especially those utilizing the Lichtenstein method, various studies have suggested that fibrin glue is an effective alternative to sutures in reducing invasiveness^1–6^. In endoscopic hernia repair, there is a growing trend towards avoiding invasiveness by not fixing the mesh, as recommended by guidelines from the HerniaSurge Group^7^. Although while unfixation is undoubtedly the most desirable approach, racial differences have not been extensively discussed. For instance, in many Japanese populations, the pelvic cavity tends to be narrow, potentially rendering a mesh size of 10 × 15 cm too large to adequately cover the myopectineal orifice (MPO)^8^. If the mesh is too small, it may fail to provide sufficient coverage of the hernia portal, necessitating fixation in practice. Sato et al. investigated cases of recurrence during laparoscopic mesh fixation and found that the mesh tended to shift ventrally at the time of recurrence, whereas cases where the mesh shifted dorsally experienced no recurrence^9^. Given that the dorsal aspect of the MPO contains vessels and nerves, which preclude tacking and suturing, fibrin glue fixation presents as a more viable option. Furthermore, several studies have indicated that fibrin glue leads to less postoperative pain compared to tacking^5,6,9–13^, potentially enhancing patient quality of life following surgery. Further, basic research comparing fixation capabilities between fibrin glue and tacks or staples and studies on meshes that are compatible with fibrin glue are advancing^14–21^. However, despite the availability of various specialized devices for fibrin glue, there are no studies assessing differences in the mesh fixation capabilities of different devices. Moreover, most clinical reports did not mention the type of device used for application. The effect of fibrin glue varies based on the type of device used for application^22–24^. This study confirmed the most effective application device for hernia mesh fixation also examined the difference in effects according to the type of mesh with the most effective application method. In this study, we evaluated the impact of different fibrin glue application methods on hernia mesh fixation capability by measuring tensile strengths in an ex vivo system.

## Methods

### Source of biological material for abdominal wall samples

The abdominal walls used in the tests were obtained from 6-week-old white Japanese male rabbits weighing 2.5–3.0 kg (purchased from KITAYAMA LABES Co., Ltd., Nagano, Japan) that were used in another animal experiment. We confirm that this study was conducted in accordance with the ARRIVE guidelines (https://arriveguidelines.org) for reporting animal research. All experimental procedures involving animals were approved by the institutional animal care and use committee of KM Biologics and was conducted in compliance with the relevant regulations for the humane treatment of laboratory animals.

### Test 1: Methods of fibrin glue application for comparing mesh fixation effects

**Figure 1:**
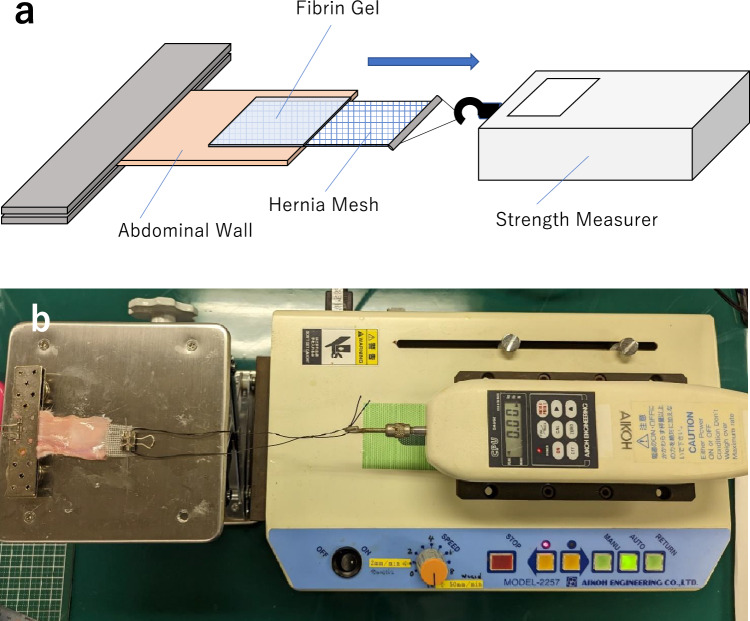
Tensile strength testing system. (**a**) The abdominal wall, extracted from a rabbit, was clamped onto a metal plate. A 2 cm square area, which is half of the trimmed hernia mesh measuring 4 × 2 cm, was fixed to the abdominal wall using fibrin glue. After a 3-min rest, mechanical traction was applied horizontally to measure the maximum tensile strength until adhesion fails. (**b**) Image of the actual testing system.

The abdominal walls were trimmed to a size of 3.0 × 6.0 cm and fixed at the edges with metal fixtures on a base. The hernia mesh (TiLENE® Mesh extralight, pfm medical titanium gmbh, Nürnberg, Germany) was trimmed to a size of 2 × 4 cm, and a loop for traction was attached to one of the ends. Half of the trimmed hernia mesh (2 cm square) closely adhered to the abdominal wall. Fibrin glue (BOLHEAL®, KM Biologics Co., Ltd., Kumamoto Japan) was applied to fix the mesh. The fibrin glue comprises two solutions (fibrinogen and thrombin). Further, 0.4 mL of each solution was used and applied using the spraying, two-liquid mixing, and sequential layering methods (Movie 1). Compressed gas or nongas types could be used in the spraying method. In this study, the nongas-type spray was selected. After 3 min of rest post-application, the mesh was pulled at a speed of 50 cm/min using a speed controller (Model-2257, Aikoh Engineering Co., Ltd., Osaka, Japan). The pulling method and its speed were based on ASTM F2458-05 (2015)^25^. The maximum tensile strength at the point of adhesion failure was measured with a strength measurer (Model-9500, Aikoh Engineering Co., Ltd., Osaka, Japan) and recorded (Fig. 1). All tests were conducted 10 times each group.

### Test 2: Effect of different types of non-heavyweight mesh on adhesive strength

**Figure 2:**
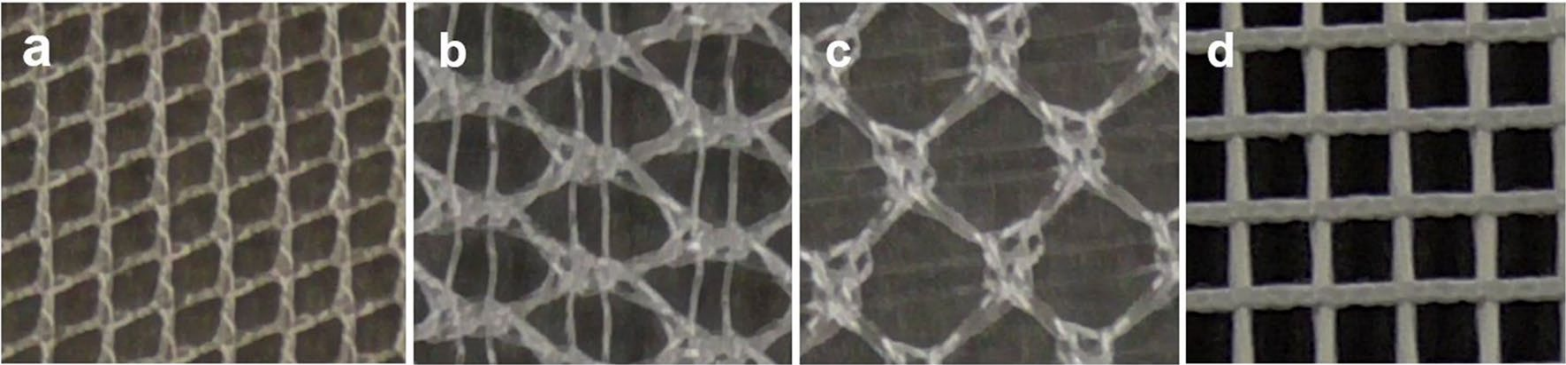
Meshes used in the test. (**a**) TiLENE® Mesh extralight. (**b**) 3DMax™ Light Mesh. (**c**) BARD® Soft Mesh. (**d**) Parietex™ Anatomical Mesh.

In addition to TiLENE® Mesh extralight, 3DMaxTM Light Mesh (BD, Warwick, Rhode Island, the United States), BARD® Soft Mesh (BD, Warwick, Rhode Island, the United States), and ParietexTM Anatomical Mesh (Medtronic plc, Minneapolis, Minnesota, the United States) were used in the same procedure as in Study 1 (Fig. 2). The spraying method using fibrin glue was adopted in this study because it had the highest adhesive strength based on the preliminary test results. All tests were conducted 10 times each group.

### Statistical analysis

In this test, the Mann–Whitney U test was used to identify significant differences. Preliminary studies showed that 10 experiments were adequate for identifying differences between the spraying, two-liquid mixing and sequential layering methods in Test 1. Statistical analysis in Test 2 was performed in an exploratory manner. Results yielding two-tailed p-values of < 0.05 were considered significant. All statistical analyses were performed using the EZR software (Saitama Medical Center, Jichi Medical University, Saitama, Japan)^26^.

## Results

### Test 1: Methods of fibrin glue application for comparing the mesh fixation effect

**Figure 3:**
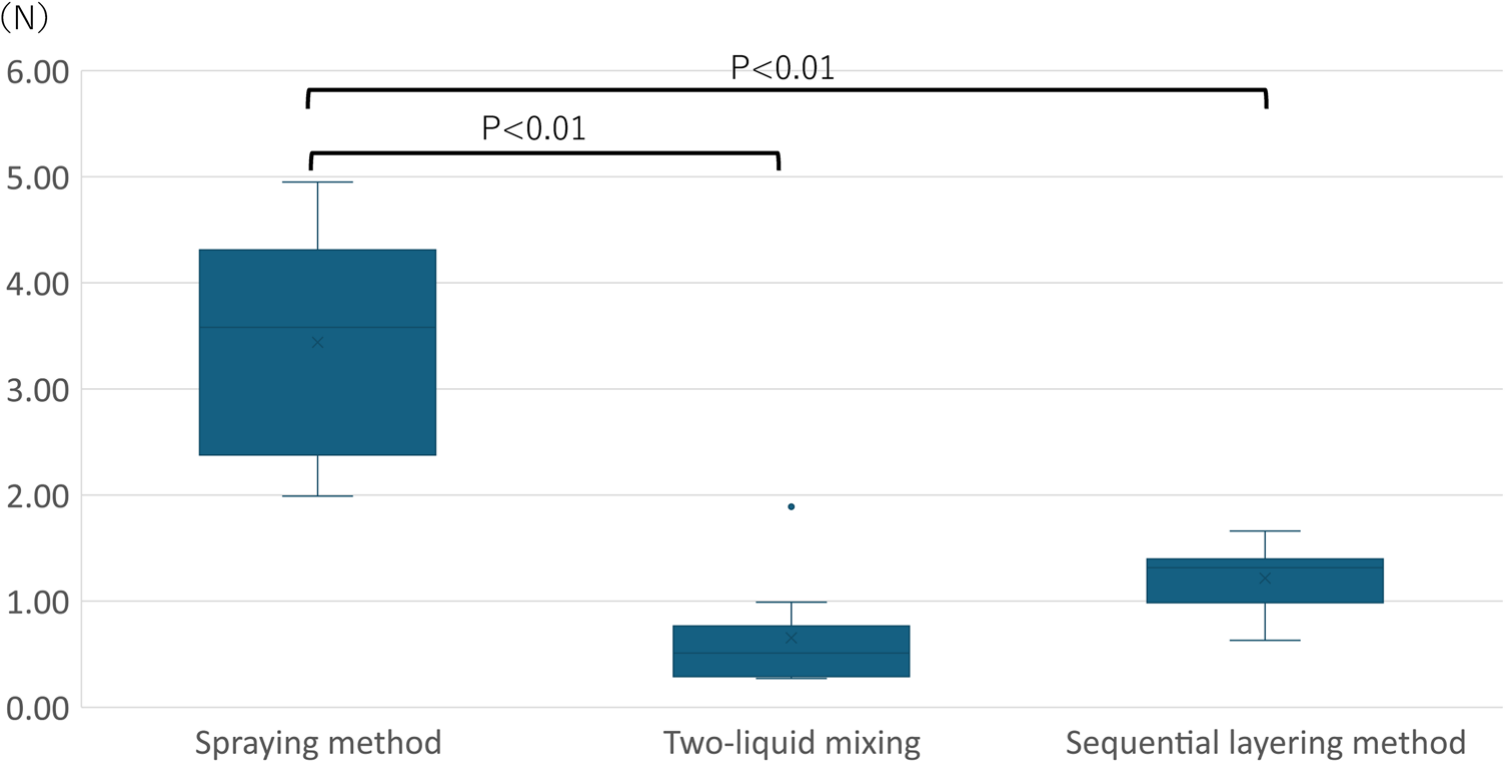
Effect of different fibrin glue application methods on mesh fixation. The spray method had predominantly higher values than the two-liquid mixing method and sequential layering method (*P* < 0.01). (*n* = 10).

The median (minimum–maximum) tensile strengths of TiLENE® Mesh extralight with the spraying, two-liquid mixing, and sequential layering methods were 3.58 (1.99–4.95), 0.51 (0.27–1.89), and 1.32 (0.63–1.66) N, respectively. The spraying method had predominantly higher tensile strength values than the two-liquid mixing and sequential layering methods (*P* < 0.01) (Fig. 3).

### Test 2: Effect of different types of non-heavyweight mesh on adhesive strength

**Figure 4:**
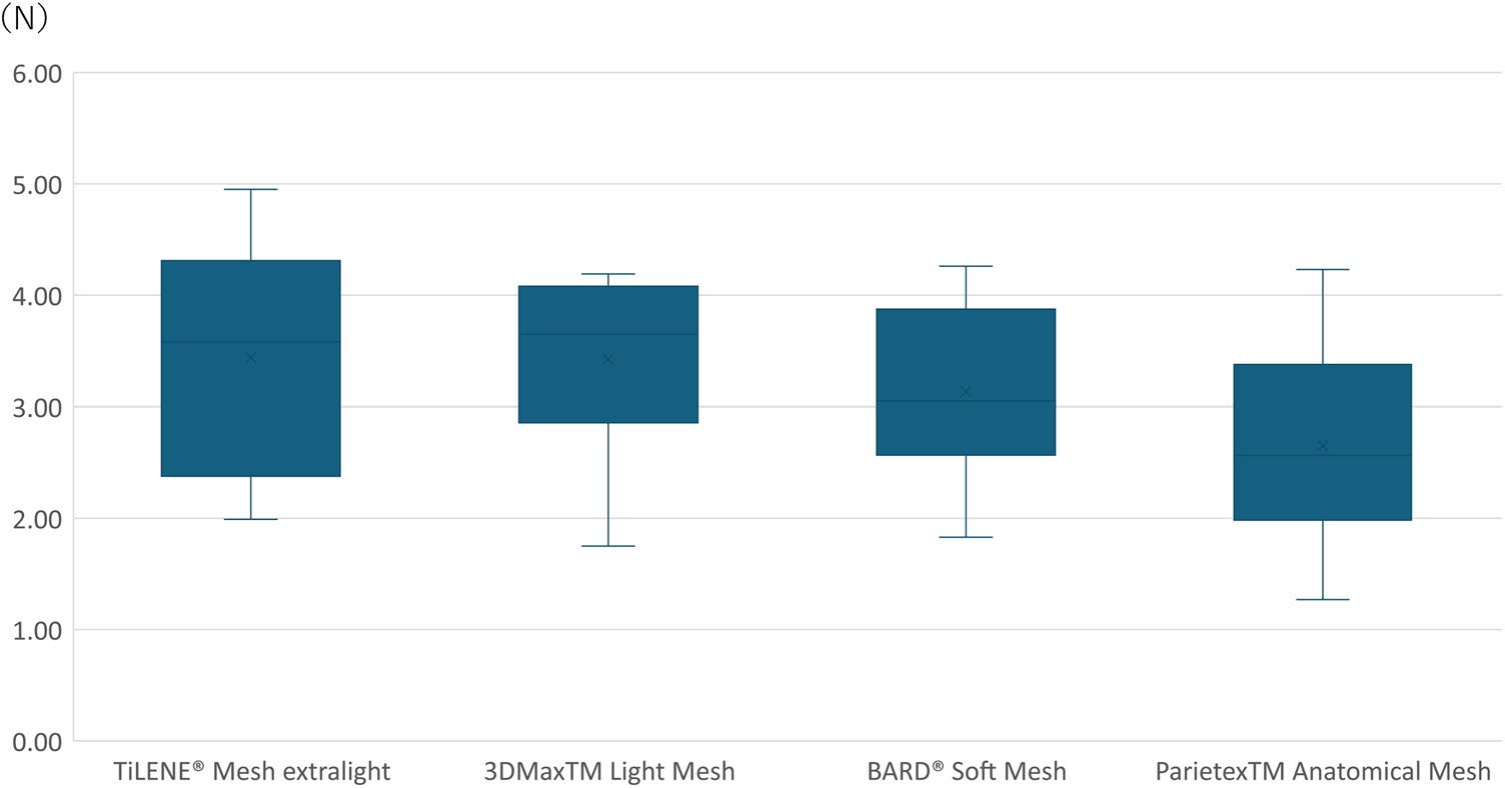
Result of fixing a nonheavy weight mesh using the fibrin glue spray method. (*n* = 10).

The median (minimum–maximum) tensile strengths of TiLENE® Mesh extralight using the spraying method was 3.58 (1.99–4.95) N. The tensile strength values of 3DMaxTM Light Mesh, BARD® Soft Mesh, and ParietexTM Anatomical Mesh were 3.65 (1.75–4.19), 3.05 (1.83–4.26), and 2.57 (1.27–4.23) N, respectively (Fig. 4). In the comparison of ParietexTM Anatomical Mesh with TiLENE® Mesh extralight and 3DMaxTM Light Mesh, it was found to have a median strength of approximately 1 N lower. However, after performing exploratory significance tests, no significant differences were detected under the conditions set, with p-values of 0.123 and 0.089, respectively.

## Discussion

The use of a spray device for fibrin glue application in hernia mesh fixation can enhance adhesive effects. This finding is in accordance with that of previous reports from other fields^22–24^, and it reconfirms the efficacy of spraying devices. The use of spraying devices also significantly reduces solution dripping when applied to slopes^24^, which is economical because it prevents fibrin glue from flowing down to unintended areas. Several studies on hernia mesh fixation with fibrin glue, including clinical and nonclinical ones, have been conducted. However, most of these studies did not specify the device used in fibrin glue application. As shown in this study, the type of device and procedure used for applying fibrin glue should be clearly specified. Unified tests on the use of fibrin glue application devices and procedures can reveal various results, potentially altering the current understanding derived from previous basic, nonclinical, and clinical research. There are two types of spray for fibrin glue. That is, one uses compressed gas, and the other utilizes a non-gas type. However, the difference in adhesive strength between the two is negligible^22^. However, caution should be taken when using compressed gas sprays in laparoscopic surgery because of the risk of gas embolism. An updated guideline on the risk of gas embolism associated with the use of fibrin glue spray was issued by the British government in 2014^27^. This guideline specifies the maximum pressure of the gas used and the minimum distance from the tissue. Further, it states that CO_2_ gas should be used for laparoscopic surgery. Experiments with pigs have reported that the spray can be safely utilized by opening the trocar valve to limit gas pressure and distance^28,29^. However, the use of compressed gas sprays should not be taken lightly, as the risk management of gas embolism is completely up to the user, except for sprays equipped with a mechanism to release the gas. Non-gas end-type sprays are not associated with a risk of gas embolism; thus, they can be used without concerns.

Moreover, the selection of mesh used in conjunction with fibrin glue was also an important factor. When fibrin glue is used for fixation, the use of a lightweight mesh with larger pores leads to a higher adhesive strength in^14^. Clinically, it reduces the consumption of analgesics for managing postoperative pain^30^. Based on these results, heavyweight meshes were preemptively excluded from this study. The four types of meshes adopted in this trial were selected with consideration of their compatibility. Thus, they have a good adhesion. However, Parietex™ Anatomical Mesh may have a slightly different adhesive strength from the other three types. That is, it is somewhat heavier and less elastic than the other three types, which might have affected compliance with the stretched rabbit abdominal wall during traction in this test. If the mesh is fixed with fibrin glue, an appropriate type of mesh should be utilized^14,21,30^, and this should be considered when optimizing the techniques.

Mesh-based hernia repair surgery is a standard procedure. Conversely, various studies on the method used for mesh fixation, including nonfixation, have been performed. As for non-fixation, it appears acceptable in many cases for endoscopic mesh placement. However, there are still instances where fixation is necessary, such as in large M3 type hernias, as outlined in the guidelines by the HerniaSurge Group^7^. Moreover, racial differences, as discussed in the introduction, may influence the efficacy of fixation. Compared with the tack-based fixation methods, the use of fibrin glue for mesh fixation is advantageous because it does not add further invasiveness to the body. Compared with endoscopic tack fixation, fibrin glue is cheaper. Moreover, in terms of postoperative management (administration of analgesics), the cost-efficacy of fibrin is higher, as shown in previous studies^11,31^. Even for groin incisions, fibrin glue is more expensive than sutures. However, when considering the reduction in surgical time, length of hospital stay, and cost of painkillers, the difference is negligible^2^.

Thus, several studies have shown that mesh fixation with fibrin glue has its advantages in cases requiring mesh fixation.. However, as indicated in the respective guidelines^7,32,33^, it is not yet considered an optimal method for mesh fixation. A meta-analysis has shown no significant difference in terms of recurrence rates between tacks and sutures^1,5,9^. However, a comparison among the reports regarding the recurrence rate in laparoscopic mesh fixation with fibrin glue reveals considerable variation,, ranging from 0 to 13.6%^8^. Thus, clinical trials have different results. However, if all trials have used fibrin glue with an optimal application method, namely, the spraying technique, the results might have been different. Several factors are associated with the recurrence of inguinal hernia^34,35^. However, a secure mesh fixation should be ensured to prevent recurrence.

To achieve a stable adhesive strength, the type of device for applying fibrin glue is important, as reconfirmed in this study. However, this was an ex vivo study using abdominal walls resected from rabbits. Therefore, future clinical trials should compare the advantages of the spraying method versus other methods in mesh fixation.

## Conclusions

The effects of fibrin glue vary significantly based on the method used. In inguinal hernia mesh fixation, the spraying method can be adopted to achieve appropriate adhesive effects. Further, when selecting a non-heavyweight mesh with excellent hydrophilicity and large pores, fibrin glue fixation with the spraying method can have stable adhesive effects.

### Supplementary Information

Supplementary Movie 1.

## Data Availability

The datasets used and/or analyzed during the current study are available from the corresponding author on reasonable request.
